# Tuberculosis co-infection and its associated factors among People living with HIV/AIDS attending antiretroviral therapy clinic in southern Ethiopia: a facility based retrospective study

**DOI:** 10.1186/s13104-018-3530-3

**Published:** 2018-06-28

**Authors:** Abel Negussie, Damene Debalke, Teshome Belachew, Fetlework Tadesse

**Affiliations:** 1 Yirgalem Hospital Medical College, Yirgalem, Ethiopia; 2 Menelik II Referral Hospital, Addis Ababa, Ethiopia

**Keywords:** Tuberculosis, HIV/AIDS, Co-infection, PLWHA, Southern Ethiopia

## Abstract

**Objective:**

The study aimed to determine the prevalence and identify determinants of TB among People living with HIV/AIDS (PLWHAs) through reviewing and analyzing patient case files from the anti-retro viral treatment (ART) clinic of Yirgalem General Hospital, southern Ethiopia.

**Results:**

Of the total PLWHAs involved in the study, 51 (36.9%) of them were found to have TB, and of which, 37 (72.5%) were smear negative cases. The multivariate analysis showed that PLWHA’s who are at WHO clinical stage 3 (AOR = 5.82; 95% CI 1.04–32.30), CD4 level of 200–500 cells/mm^3^ (AOR = 4.85; 95% CI 1.95–12.05) and < 200 cells/mm^3^ (AOR = 7.34; 95% CI 2.75–19.58) at ART initiation, and who didn’t take INH prophylaxis (AOR = 12.36; 95% CI 4.47–34.14) were significantly associated with TB-HIV co-infection. Rapid and sensitive diagnostic techniques should be implemented to early detect co-infections, and also INH prophylactic preventive measures should be strengthened to reduce TB incidence.

**Electronic supplementary material:**

The online version of this article (10.1186/s13104-018-3530-3) contains supplementary material, which is available to authorized users.

## Introduction

Tuberculosis (TB) remains a major public health problem in resource-limited countries and it is the largest cause of death among People living with HIV/AIDS (PLWHAs) worldwide. According to the WHO Global TB Report, there were an estimated 9 million new cases of TB occurred worldwide, of which 13% were associated with HIV/AIDS infection, and 78% of these HIV/AIDS associated cases occurred in Africa. It is further estimated that 1.49 million deaths from TB, including 0.36 million among PLWHAs, and 96% of the deaths occurred in developing countries. Ethiopia has been classified 7th among the 22 high burden countries with TB and HIV/AIDS infection in the world [1, 2].

HIV/AIDS predisposes for Mycobacterium tuberculosis (MTB) infection and increases the probability of recently acquired TB infection to progress to the status of active disease and increases the rate of recurrent TB. The lifetime risk of developing active TB in immune-competent adults is estimated to be 10%, but in PLWHAs who infected with MTB the annual risk of developing active TB disease exceeds 10% [3].

The current increasing HIV/AIDS associated tuberculosis shifted the clinical pattern of TB towards smears negative pulmonary TB (PTB) and extra-pulmonary TB (EPTB), which in turn, causes difficulties in the diagnosis and treatment of TB due to unusual clinical picture with increased smear negative acid fast Bacilli (AFB) PTB, atypical finding on chest radiography and increased prevalence of EPTB [4, 5].

Several studies documented that the clinical manifestations of TB in PLWHAs are quite varied and generally show different patterns as a function of the CD4+ T cell count. In addition, TB can appear at any stage of HIV/AIDS infection, and its presentation varies with the WHO stage and CD4+ lymphocyte count, although it is more frequent at CD4+ T cell count of < 300 cells/mm^3^ [6, 7].

In the study area, there is no adequate and recent data on the prevalence and determinants of TB in PLWHAs. Even though it is expected that the prevalence of TB among PLWHAs in the study area is assumed to be higher than other sites in the region due to the large number of PLWHAs treated in the ART clinic, this expectation should be supported by area specific study. The objective of the study was to determine the prevalence of TB and its determinant factors among PLWHAs in a local context that will help to reduce the burden of the disease by facilitating the early detection of at-risk patients and to serve as a reference document for further studies.

## Main text

### Methods

The study was conducted in Yirgalem General Hospital, which is found in Yirgalem town, Sidama Zone, southern Ethiopia. It serves around 4.2 million populations of Sidama Zone and part of southern Oromia region. A total of 5944 PLWHAs were registered since the ART clinic in the hospital had started its function in 2001. The trend of HIV/AIDS cases has decreased over time and the highest number of cases was registered in 2005/2006 with a total of 1219 cases.

A facility-based retrospective cross-sectional study was carried out to determine the prevalence of TB and identify its determinant factors among PLWHAs through reviewing and analyzing a 2 year (from January 1, 2015 to December 30, 2016) patient case files from the ART clinic of Yirgalem General Hospital. All PLWHAs attending the ART clinic of Yirgalem General Hospital during the study period were eligible for the study, and a total of 182 PLWHAs were diagnosed and treated during the period.

Taking the estimated proportion (P) of TB among PLWHAs = 0.1 [1], margin of error = 5% and confidence level = 95%; the calculated sample size became 138. The 2 years HIV/AIDS patients’ case files were identified and the study participants were selected by lottery method after excluding patients with incomplete medical records.

Data was collected from patients’ case files using a checklist which was prepared by adopting previous studies [6–8]. The checklist was pre-tested before the actual data collection and some variables like adherence to ART medications and nutritional status were omitted because of incomplete medical records. Otherwise, the checklist was inclusive of the different variables (age, sex, occupation, residence, INH prophylaxis, CD4 count, ART status) which were assumed as possible determinants of TB occurrence among PLWHAs.

The data were entered, cleaned and analyzed using SPSS version 19 statistical software. Frequencies and percentages were calculated for descriptive analysis of socio-demographic, clinical and laboratory profile of the study participants. Binary and multiple logistic regression analyses were applied to determine associations and identify risk factors of TB occurrence among PLWHAs taking P ≤ 0.05 for statistical significance.

### Results

A total of 138 PLWHA’s medical records were reviewed and most of the participants were females (57.9%), aged 16–45 years (83.3%), urban residents (56.5%) and Protestants (52.2%) by religion. Moreover, 65 (48.9%) of the study participants were married and 73 (54.9%) of them attended primary school (Additional file 1: Table S1).

**Fig. 1:**
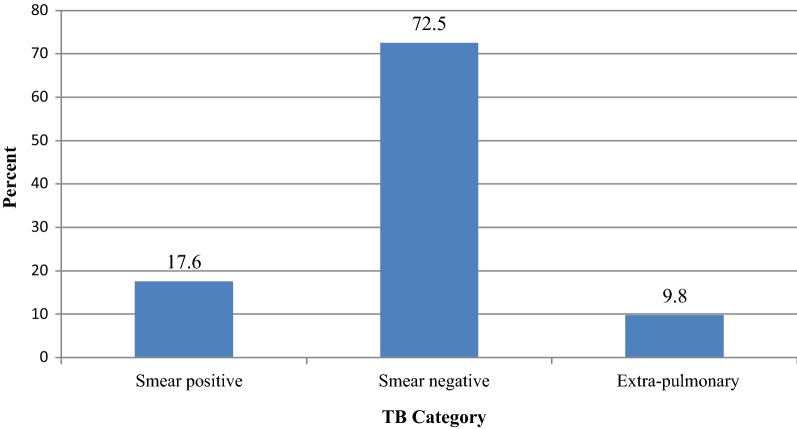
Types of TB among PLWHAs, Yirgalem General Hospital, southern Ethiopia; January 2015 to December 2016.  TB category

Of the total PLWHAs involved in the study, 51 (36.9%) of them were found to have TB; and of the 51 with TB, 37 (72.5%) were smear negative cases (Fig. 1). In addition, 37 (72.5%), 9 (17.6%) and 3 (5.9%) of the TB positive patients were diagnosed by chest x-ray, microscopy (AFB) and biopsy, respectively.

Out of the total selected PLWHAs, 47 (34%) of them didn’t take INH prophylaxis; and among them, 35 (74.5%) were TB positive patients. Furthermore, 126 (91.3%) of the study participants had started ART, and of them 61 (48.4%) had CD4 level of < 200 cells/mm^3^ at ART initiation (Table 1).

**Table 1:** Clinical and laboratory profiles of study participants in Yirgalem General Hospital, southern Ethiopia; January 2015 to December 2016

Variables	Category	Frequency	Percent
TB diagnosis	Positive	51	37.0
	Negative	87	63.0
	Total	138	100
INH prophylaxis	Yes	91	65.9
	No	47	34.1
	Total	138	100
WHO stage	Stage 1	66	47.8
	Stage 2	30	21.7
	Stage 3	29	21.0
	Stage 4	13	9.4
	Total	138	100
ART status	Pre-ART	12	8.7
	ART	126	91.3
	Total	138	100
CD4 level at ART initiation	> 500	21	16.7
	200–500	44	34.9
	< 200	61	48.4
	Total	126	100
CD4 level during diagnosis	> 500	27	19.9
	200–500	49	36.0
	< 200	60	44.1
	Total	136	100

The bivariate analysis result showed that residence, INH prophylaxis status, WHO clinical stage and CD4 level at ART initiation were associated with TB-HIV/AIDS co-infection. In the multivariate analysis, multiple logistic regression was used to identify determinants of TB occurrence among PLWHAs. Being at WHO clinical stage 3 (AOR = 5.82; 95% CI 1.04–32.30), not taking INH prophylaxis (AOR = 12.36; 95% CI 4.47–34.14), CD4 level of < 200 cells/mm^3^ (AOR = 7.34; 95% CI 2.75–19.58) and a CD4 level of 200–500 cells/mm^3^ (AOR = 4.85; 95% CI 1.95–12.05) at ART initiation were significant determinants of increased risk of TB co-infection among PLWHAs (Table 2).

**Table 2:** Determinant factors of TB-HIV co-infection among PLWHAs, Yirgalem General Hospital, southern Ethiopia, 2017

Variables	TB disease		COR (95% CI)	AOR (95% CI)	*P* value
	Yes	No			
Age (years)					
< 15	2	3	1.12 (1.16–9.01)		
16–45	41	74	1.44 (0.52–9.94)		
≥ 46	8	10	1		
Sex					
Male	21	37	1.05 (0.52–2.13)		
Female	30	50	1		
Residence					
Urban	24	54	1	1	
Rural	27	33	0.54 (0.27–1.09)	0.65 (0.23–1.77)	0.40
Occupation					
Housewife	18	31	1		
Government employee	4	8	1.16 (0.30–4.40)		
Merchant	9	12	0.77 (0.27–2.19)		
Farmer	6	11	1.06 (0.33–3.36)		
Student	5	4	0.46 (0.11–1.95)		
Daily laborer	3	2	0.38 (0.05–2.54)		
Unemployed	3	1	1.19 (0.01–2.00)		
Marital status					
Married	26	47	1		
Single	10	17	0.94 (0.37–2.35)		
Divorced	11	15	0.75 (0.30–1.88)		
Widowed	3	4	0.73 (0.5–3.55)		
INH prophylaxis					
Yes	16	75	1	1	
No	35	12	13.67 (5.84–31.96)	12.36 (4.47–34.14)	< 0.01*
ART profile					
Pre-ART	3	9	1.84 (0.47–7.15)		
ART	48	78	1		
WHO stage					
Stage 1	0	66	1	1	
Stage 2	10	20	0.61 (0.15–2.42)	0.74 (0.12–4.32)	0.16
Stage 3	21	8	7.20 (2.00–25.90)	5.82 (1.04–32.30)	0.002*
Stage 4	8	5	3.20 (0.82–12.35)	2.10 (0.35–12.37)	0.06
CD4 level at ART initiation					
> 500	5	16	1	1	
200–500	13	31	2.30 (1.01–5.23)	4.85 (1.95–12.05)	< 0.01*
< 200	30	31	3.09 (1.00–9.51)	7.34 (2.75–19.58)	< 0.01*

* Statistically significant at P < 0.05

### Discussion

Tuberculosis is a well-recognized opportunistic infection in patients with HIV/AIDS. In our study, we found that TB-HIV/AIDS co-infection was 36.9% and it was higher than the findings of other studies [5, 8–10]. This variation in the magnitude of tuberculosis in PLWHAs may be due to high TB detection rate as a result of availability of TB-diagnostic facilities or due to differences in HIV/AIDS infection rate at the population level.

The other relevant finding is that 72.5% of the TB-HIV/AIDS co-infected cases were smear-negative and this result is higher than the findings of other studies conducted elsewhere [7, 8]. This higher smear negativity indicates the importance of TB diagnostic techniques, other than AFB microscopy. In the study area, clinical data is supplemented with chest radiography to diagnose suspicious smear negative cases.

INH prophylaxis status, being at WHO clinical stage of 3, having a CD4 level of 200–500 and < 200 cells/mm^3^ at ART initiation were identified as risk factors of TB occurrence among PLWHAs. These findings are in line with other Hospital-based studies in Ethiopia [7, 8].

Our study revealed a poor INH prophylactic management for PLWHAs. But, the role of INH prophylaxis in the reduction of TB incidence has been reported in many clinical trials and found to be important [3]. The most important determinant factor for development of TB in PLWHAs is the immunologic state of the person and maintaining the CD4+ lymphocyte cell count level as high as possible helps the person to have a low risk of infection.

### Conclusions

In our study, the prevalence of TB and the proportion of smear negative TB among PLWHAs was high. The risk of TB occurrence was found to be high among PLWHAs with WHO clinical stage 3, low CD4 level at ART initiation and PLWHAs who didn’t receive INH prophylaxis.

The high proportion of smear negative TB cases among PLWHAs requires special attention for confirming clinically suspected TB cases; and in addition to clinical and radiological investigations, rapid and sensitive diagnostic techniques like Culture, Fluorescent Microscopy and Xpert MTB/RIF should be implemented by Yirgalem General Hospital. Furthermore, our study revealed a poor INH prophylactic management for PLWHAs, and therefore, INH prophylactic preventive measures should be strengthened in the Hospital to reduce TB incidence among PLWHAs.

## Limitations

Despite the study’s endeavor of identifying determinants of TB infection among PLWHAs, as the study is a facility based study undertaken in a single hospital with limited number of patients, it is difficult to make generalizations and future community-based studies are needed to substantiate the study findings.

## Additional file

**Additional file 1: Table S1.** Socio-demographic profile of study participants in Yirgalem General Hospital, southern Ethiopia; January 2015 to December 2016.

## Abbreviations

AFB: acid fast Bacilli
AOR: adjusted odds ratio
ART: anti-retro viral treatment
EPTB: extra-pulmonary tuberculosis
MTB: Mycobacterium tuberculosis
PLWHA: People living with HIV/AIDS
PTB: pulmonary tuberculosis
TB: tuberculosis
WHO: World Health organization
