# Supplementary material for: Tuberculosis co-infection and its associated factors among People living with HIV/AIDS attending antiretroviral therapy clinic in southern Ethiopia: a facility based retrospective study
Source: BMC Res Notes. 2018 Jun 28;11:417. doi: 10.1186/s13104-018-3530-3 (PMC6025838; doi:10.1186/s13104-018-3530-3)
Supplement: Supplementary file 1 — Additional file 1: Table S1. Socio-demographic profile of study participants in Yirgalem General Hospital, southern Ethiopia; January 2015 to December 2016. [file 13104_2018_3530_MOESM1_ESM.doc]

**Table S1: Socio-demographic profile of study participants in Yirgalem General Hospital, southern Ethiopia; January 2015 to December 2016**

| Variable | Category | Frequency | Percent |
| --- | --- | --- | --- |
| Age | < 15yrs | 5 | 3.6 |
|  | 16-45yrs | 115 | 83.3 |
|  | ≥46yrs | 18 | 13 |
|  | Total | 138 | 100 |
| Sex | Male | 58 | 42.0 |
|  | Female | 80 | 58.0 |
|  | Total | 138 | 100 |
| Occupation | House wife | 49 | 41.2 |
|  | Government employee | 12 | 10.1 |
|  | Merchant | 21 | 17.6 |
|  | Farmer | 17 | 14.3 |
|  | Student | 9 | 7.6 |
|  | Daily laborer | 5 | 4.2 |
|  | Unemployed | 4 | 3.4 |
|  | Total | 117 | 100 |
| Religion | Protestant | 72 | 52.2 |
|  | Orthodox | 59 | 42.8 |
|  | Muslim | 5 | 3.6 |
|  | Catholic | 2 | 1.4 |
|  | Total | 138 | 100 |
| Residence | Urban | 78 | 56.5 |
|  | Rural | 60 | 43.5 |
|  | Total | 138 | 100 |
| Marital Status | Married | 73 | 54.9 |
|  | Single | 27 | 20.3 |
|  | Divorce | 26 | 19.5 |
|  | Widow | 7 | 5.3 |
|  | Total | 133 | 100 |
| Educational Status | Illiterate | 21 | 15.8 |
|  | Primary School | 65 | 48.9 |
|  | Secondary School | 39 | 29.3 |
|  | Tertiary | 8 | 6.0 |
|  | Total | 133 | 100 |
